# Mouse aorta-derived mesenchymal progenitor cells contribute to and enhance the immune response of macrophage cells under inflammatory conditions

**DOI:** 10.1186/s13287-015-0071-8

**Published:** 2015-04-14

**Authors:** Jodi F Evans, Veronica Salvador, Sheela George, Cristina Trevino-Gutierrez, Catherine Nunez

**Affiliations:** Biomedical Research Core, Winthrop University Hospital, 222 Station Plaza North, Mineola, NY 11501 USA; Stony Brook University School of Medicine, 222 Station Plaza North Suite 501, Mineola, NY 11501 USA; Molloy College, 1000 Hempstead Avenue, Rockville Centre, NY 11571 USA

## Abstract

**Introduction:**

Mesenchymal progenitor cells interact with immune cells and modulate inflammatory responses. The cellular characteristics required for this modulation are under fervent investigation. Upon interaction with macrophage cells, they can contribute to or suppress an inflammatory response. Current studies have focused on mesenchymal progenitors derived from bone marrow, adipose, and placenta. However, the arterial wall contains many mesenchymal progenitor cells, which during vascular disease progression have the potential to interact with macrophage cells. To examine the consequence of vascular-tissue progenitor cell-macrophage cell interactions in an inflammatory environment, we used a recently established mesenchymal progenitor cell line derived from the mouse aorta.

**Methods:**

Mouse bone marrow-derived macrophage (MΦ) cells and mouse aorta-derived mesenchymal progenitor (mAo) cells were cultured alone or co-cultured directly and indirectly. Cells were treated with oxidized low-density lipoprotein (ox-LDL) or exposed to the inflammatory mediators lipopolysaccharide (LPS) and interferon-gamma (IFNγ) or both. A Toll-like receptor-4 (TLR4)-deficient macrophage cell line was used to determine the role of the mAo cells. To monitor inflammation, nitric oxide (NO), interleukin-6 (IL-6), and tumor necrosis factor-alpha (TNFα) secretions were measured.

**Results:**

Mesenchymal progenitor cells isolated from aorta and cloned by high proliferative capacity (mAo) can differentiate into multiple mesenchymal lineages and are positive for several commonly used mouse mesenchymal stem cell markers (that is, CD29, CD44, CD105, CD106, and Sca-1) but are negative for CD73 and ecto-5′-nucleotidase. In co-culture with MΦ cells, they increase MΦ oxidized-LDL uptake by 52.2%. In an inflammatory environment, they synergistically and additively contribute to local production of both NO and IL-6. After exposure to ox-LDL, the inflammatory response of MΦ cells to LPS and LPS/IFNγ is muted. However, when lipid-laden MΦ cells are co-cultured with mAo cell progenitors, the muted response is recovered and the contribution by the mAo cell progenitor is dependent upon cell contact.

**Conclusions:**

The resident mesenchymal progenitor cell is a potential contributor to vascular inflammation when in contact with inflamed and lipid-laden MΦ cells. This interaction represents an additional target in vascular disease treatment. The potential for resident cells to contribute to the local immune response should be considered when designing therapeutics targeting inflammatory vascular disease.

**Electronic supplementary material:**

The online version of this article (doi:10.1186/s13287-015-0071-8) contains supplementary material, which is available to authorized users.

## Introduction

Mesenchymal progenitor cells have the capacity for tissue repair through direct differentiated cell replacement and also the ability to regulate immune responses during inflammation [1,2]. In immune studies, mesenchymal progenitors isolated from bone marrow, adipose tissue, and placenta have received the most attention. These progenitor populations can suppress T-cell proliferation, induce regulatory T cells, and promote the differentiation of the anti-inflammatory macrophage [3-5]. However, mesenchymal stem cells (MSCs) and progenitor cells are present in the arterial wall [6] and the role these tissue-specific cells play in vascular inflammation and disease remains unclear [7].

During vascular inflammation, monocytes enter the artery wall in response to activated endothelium and differentiate into macrophages. Macrophage cells that have entered the sub-endothelium play a role in both inflammation and resolution of inflammation in the vasculature [8]. The traditionally activated macrophage (M1), differentiated in the presence of inflammatory mediators such as lipopolysaccharide (LPS) and interferon-gamma (IFNγ), is pro-inflammatory and contributes to local production of inflammatory cytokines such as interleukin-12 (IL-12), tumor necrosis factor-alpha (TNFα), and IL-6 [9]. Macrophage cells also ingest lipoproteins in the form of oxidized low-density lipoprotein (ox-LDL) that have been retained in the sub-endothelium. These lipid-laden macrophage cells or ‘foam cells’ are associated with an inflammatory response that leads to the attraction of additional monocytes as well as T cells and mast cells [8]. However, the alternatively activated macrophage phenotype (M2) is associated with increased expression of anti-inflammatory cytokines such as IL-10 and acts in resolution of inflammation and tissue repair [10,11].

Like macrophage cells, mesenchymal progenitor cells can experience phenotypic polarization and display an immunosuppressive or pro-inflammatory phenotype [12]. Immunosuppressive mesenchymal progenitor cells promote a ‘switch’ in the macrophage cell phenotype from the inflammatory M1 to the anti-inflammatory M2 [3-5]. Conversely, some studies report that mesenchymal progenitors display a pro-inflammatory phenotype when cultured with macrophage cells [13]. During their differentiation, sub-endothelial macrophages and foam cells come in contact with the many mesenchymal progenitors in the arterial wall. Here, we sought to determine whether the interaction between aorta-derived mesenchymal progenitor cells and macrophages has the potential to contribute to or suppress inflammation in an environment associated with vascular disease.

Mouse bone marrow-derived macrophage (MΦ) cells and a recently established mouse aorta-derived mesenchymal progenitor (mAo) cell line [14] were cultured alone or co-cultured directly and indirectly. The cells were treated with ox-LDL or exposed to the inflammatory mediators LPS and IFNγ or both. A Toll-like receptor-4-deficient macrophage (TLR4-MΦ) cell line was used to determine the role of the mAo cells in the inflammatory response. To monitor inflammation, nitric oxide (NO), IL-6, and TNFα secretions were measured.

## Methods

### Materials

All cell culture media, trypsin, fetal bovine serum (FBS), and antibiotic/antimycotic solutions were obtained from Invitrogen (Carlsbad, CA, USA). The L-929 fibroblast (CCl-1), LADMAC macrophage/monocyte (CRL-2420), and C3H/HeJ mouse I-13.35 splenic cell lines (CRL-2471) deficient for TLR-4 were purchased from the American Type Tissue Collection (ATCC) (Manassas, VA, USA). Endotoxin tested (less than 0.1 ng/μg) IFNγ (#I1000) was purchased from US Biological (Salem, MA, USA), the inducible nitric oxide synthase (iNOS) (#2977) antibody from Cell Signaling Technology (Danvers, MA, USA), the anti-monocyte/macrophage antibody (MOMA-2, ab33451) and anti-integrin beta-1 antibody (CD29) (#ab23834) from Abcam (Cambridge, MA, USA), and the fluorescein isothiocyanate (FITC) rat anti-mouse CD44 (#553133), phycoerythrin (PE) rat anti-mouse CD105 (#562759), PE rat anti-mouse Ly-6AE (Sca-1) (#561076), FITC rat anti-mouse CD45 (#553080), FITC rat anti-mouse CD106 (#553332), PE rat anti-mouse CD73 (#557041), and FITC rat anti-mouse CD11b (#553310) were purchased from BD Biosciences (San Jose, CA, USA). Human ox-LDL was purchased from Intracel (Frederick, MD, USA). Gamma-irradiated LPS from *Escherichia coli* (#L4391) and all other chemicals and reagents were purchased from Sigma-Aldrich (St. Louis, MO, USA) unless otherwise specified.

### Animals

All animal protocols were approved by the Winthrop University-Hospital Animal Care and Use Committee and adhere to the regulations outlined by the National Institutes of Health. C57BL/6 male mice were obtained from Taconic (Hudson, NY, USA). Animals were housed under local vivarium conditions (12-hour light-dark cycle) and allowed to acclimate for at least 7 days prior to experimentation. Mice were euthanized under CO_2_ at 8 to 12 weeks of age, and aorta and hind limbs were removed in preparation for cell isolation.

### Cell isolation and culture

#### Aortic mesenchymal progenitor (mAo) cell line

Mouse aortic progenitor cells were derived from the C57BL/6 mouse by using the method of da Silva and colleagues [6] with slight modification as described in [14].

#### Bone marrow-derived macrophage (MΦ) cells

Bone marrow from the hind limbs of the C57BL/6 mouse was isolated as previously described [15]. After a single-cell suspension was created, nucleated cells were counted by using 3% acetic acid/trypan blue exclusion and plated in Dulbecco’s modified Eagle’s medium (DMEM) supplemented with 10% FBS, 15% L929 fibroblast cell conditioned medium, 100 U/mL penicillin sodium, 100 U/mL streptomycin sulfate, and 0.25 μg/mL amphotericin B at 10^7^ cells per 100-mm petri dish. The L929 conditioned medium was prepared as suggested by the ATCC. The L-929 cell line produces macrophage colony-stimulating factor (M-CSF), which supports the growth and differentiation of macrophages from the bone marrow. After 3 days, half the medium was removed and replaced with fresh medium. A complete medium change was performed on day 6. At day 7 of culture, monocyte/macrophage cells were re-plated according to experimental objectives, passed or frozen, and stored in liquid nitrogen (LN_2_). Cultures up to passage 2 were used in experiments.

#### C3H/HeJ mouse I-13.35 splenic macrophage (TLR4-MΦ) cell line

The I-13.35 cell line is deficient in TLR-4 and was maintained in DMEM supplemented with 10% FBS, 20% LADMAC cell conditioned medium, 100 U/mL penicillin sodium, 100 U/mL streptomycin sulfate, and 0.25 μg/mL amphotericin B. The LADMAC conditioned medium was prepared as suggested by the ATCC. The LADMAC cells produce colony-stimulating factor 1 (CSF-1)/M-CSF which supports the growth of the TLR4-MΦ cell line. Cultures were initiated at 10^5^ per mL in 24-well plates or 60-mm dishes with and without a confluent layer of mAo cells according to experimental objectives.

#### Co-culture

The mAo cells were initiated at a density of 1.5 × 10^4^/cm^2^ and allowed to reach confluence. MΦ or TLR4-MΦ cells were added at a density of 1.0 × 10^5^/cm^2^ and allowed to attach overnight. Cultures were left untreated, treated with LPS (100 ng/mL), or treated with both LPS and IFNγ (250 ng/mL) for 24 hours. Some cultures were exposed to ox-LDL (50 μg/mL) overnight prior to exposure to inflammatory mediators. Culture supernatants were collected and stored at −80°C until assay.

#### Transwell cultures

For assays using transwell culture, MΦ cells were seeded at a density of 1.0 × 10^5^/cm^2^ in a transwell insert with a 0.4-μm pore-size filter and treated 24 hours with or without 50 μg/mL ox-LDL. mAo MSCs were seeded in the well at a density of 1.5 × 10^4^/cm^2^. Media in both the insert and well were left untreated, treated with LPS (100 ng/mL), or treated with both LPS and IFNγ (250 ng/mL) for 24 hours. Media from both insert and well were collected and assayed separately.

#### Conditioned medium cultures

For conditioned medium experiments, the conditioned medium was collected from mAo or MΦ cell cultures that had been treated with ox-LDL (50 μg/mL) for 24 hours and then treated with LPS (100 ng/mL) or treated with both LPS and IFNγ (250 ng/mL) for 24 hours. The conditioned medium was incubated with mAo or MΦ cell cultures that had been exposed to ox-LDL. After 24 hours, the incubated medium was collected, and NO, IL-6, and TNFα were measured. NO, IL-6, and TNFα present in the conditioned medium were subtracted from the incubated medium values to obtain the net production.

### Flow cytometry

For staining of mAo cell surface antigens, single-cell suspensions were first incubated with Fc receptor blocking reagent (Miltenyi Biotech, Bergisch Gladbach, Germany) followed by staining with antibodies against surface markers CD11b, CD29, CD44, CD45, CD73, CD105, CD106, Sca-1 (Ly-6AE), and CD45 for 30 minutes at 4°C. After incubation, cells were washed twice in wash buffer (1% FBS in phosphate-buffered saline, or PBS) and analyzed with an Accuri C6 flow cytometer (BD Biosciences).

For intracellular MOMA-2 staining of monocyte/macrophage cultures, cells were fixed and permeabilised by using the Fix and Perm Kit (Invitrogen) in accordance with the protocol of the manufacturer. Briefly, the cells were fixed with buffer A for 15 minutes at room temperature and washed once with wash buffer. Fixed cells were permeabilised with buffer B for 20 minutes at room temperature, and intracellular staining antibodies were added simultaneously. After incubation, cells were washed twice in wash buffer and analyzed with the flow cytometer. Flow cytometry data was analyzed by using FlowJo software (Tree Star Inc., Ashland, OR, USA).

### Oil Red O staining

After fixing in 10% phosphate-buffered formalin, cultures were rinsed in PBS, air-dried, and stained with Oil Red O (0.5 g in 100 mL isopropanol) for 15 minutes. Wells were extensively washed with dH_2_O followed by imaging and removal of stain by using 100% isopropanol. The amount of stain was quantified by using spectrophotometry at a 550-nm wavelength.

### Alcian blue/von Kossa staining

mAo cells were grown in chondrogenic medium—DMEM supplemented with 1% insulin-transferrin-selenium, 40 μg/mL L-proline, 50 μg/mL ascobate-2-phosphate, 10 ng/mL transforming growth factor-beta 1 (TGFβ1), 100 U/mL penicillin sodium, 100 U/mL streptomycin sulfate, and 0.25 μg/mL amphotericin B—for 28 days before fixing in 10% PBS. Cultures were stained for mineral by using von Kossa’s method followed by proteoglycan staining with Alcian Blue.

### Nitrite measurements

Nitrite, as a reflection of NO production, was measured in cell culture supernatant by using the Griess Reagent system (Promega, Madison, WI, USA) in accordance with the instructions of the manufacturer.

### Western blotting

Whole lysates were prepared from cultures that had been left untreated, treated with LPS (100 ng/mL), or treated with both LPS and IFNγ (250 ng/mL) for 24 hours. Protein concentrations were determined by bicinchoninic acid assay. Protein samples (50 μg per lane) were separated by SDS-PAGE and transferred to polyvinylidene fluoride membrane. After blocking in 5% milk in Tris-buffered saline with 0.1% tween-20 (TBST), membranes were incubated overnight with iNOS (#2977) primary antibody from Cell Signaling Technology (Danvers, MA, USA) diluted 1:500 with 5% bovine serum albumin (BSA) in TBST. After incubation with anti-rabbit IgG horseradish peroxidase-tagged secondary antibody from Santa Cruz Biotechnology (Santa Cruz, CA, USA), bands were visualized by using enhanced chemiluminescence. To normalize protein loading, blots were also probed for β-actin expression.

### Secreted cytokine and chemokine measurements

The Proteome Profiler Array, mouse cytokine array panel A, from R&D Systems (Minneapolis, MN, USA) was used to screen for changes in secreted cytokines and chemokines in MΦ cells and co-cultures with and without treatment with ox-LDL. TNFα and IL-6 were measured in culture supernatants by using Ready-set-Go enzyme-linked immunosorbent assay (ELISA) kits from eBioscience (San Diego, CA, USA).

### Statistical analyses

Data was analyzed by using two-way analysis of variance. *Post hoc* test *P* values were adjusted by using the Bonferroni correction. All tests were two-tailed, and a nominal significance level of 0.05 was used.

## Results

### Characterization of the aorta-derived mesenchymal progenitor (mAo) cells and the bone marrow-derived macrophage (MΦ) cells

Mesenchymal progenitor cells were previously isolated from mouse aorta (mAo) and cloned by high proliferative capacity. We have demonstrated their ability to undergo osteoblast and adipocyte differentiation and their high expression of vimentin, fibronectin, and β1-integrin (CD29) [14]. To characterize these cells further, we used flow cytometry to examine their expression of multipotent stromal cells also known as MSC-associated cell surface markers and tested their ability to differentiate into chondrocytes. The mAo cells express the MSC-associated cell surface antigens CD29, CD44, CD105, CD106, and Sca-1 but not CD73 and are capable of mineralizing chondrogenic differentiation as demonstrated by positive Alcian Blue/von Kossa staining. They are negative for the hematopoietic cell surface antigens CD11b and CD45 (Additional file 1: Figure S1A and C). To confirm that our macrophage isolation procedure produces a homogeneous population of cells, we demonstrate that they express the monocyte/macrophage intracellular marker MOMA-2 and are resistant to sodium fluoride inhibition of non-specific esterase stain (Additional file 1: Figure S1B and C).

### mAo cells in culture with MΦ cells contribute to local NO production in response to LPS and LPS/IFNγ

**Figure 1:**
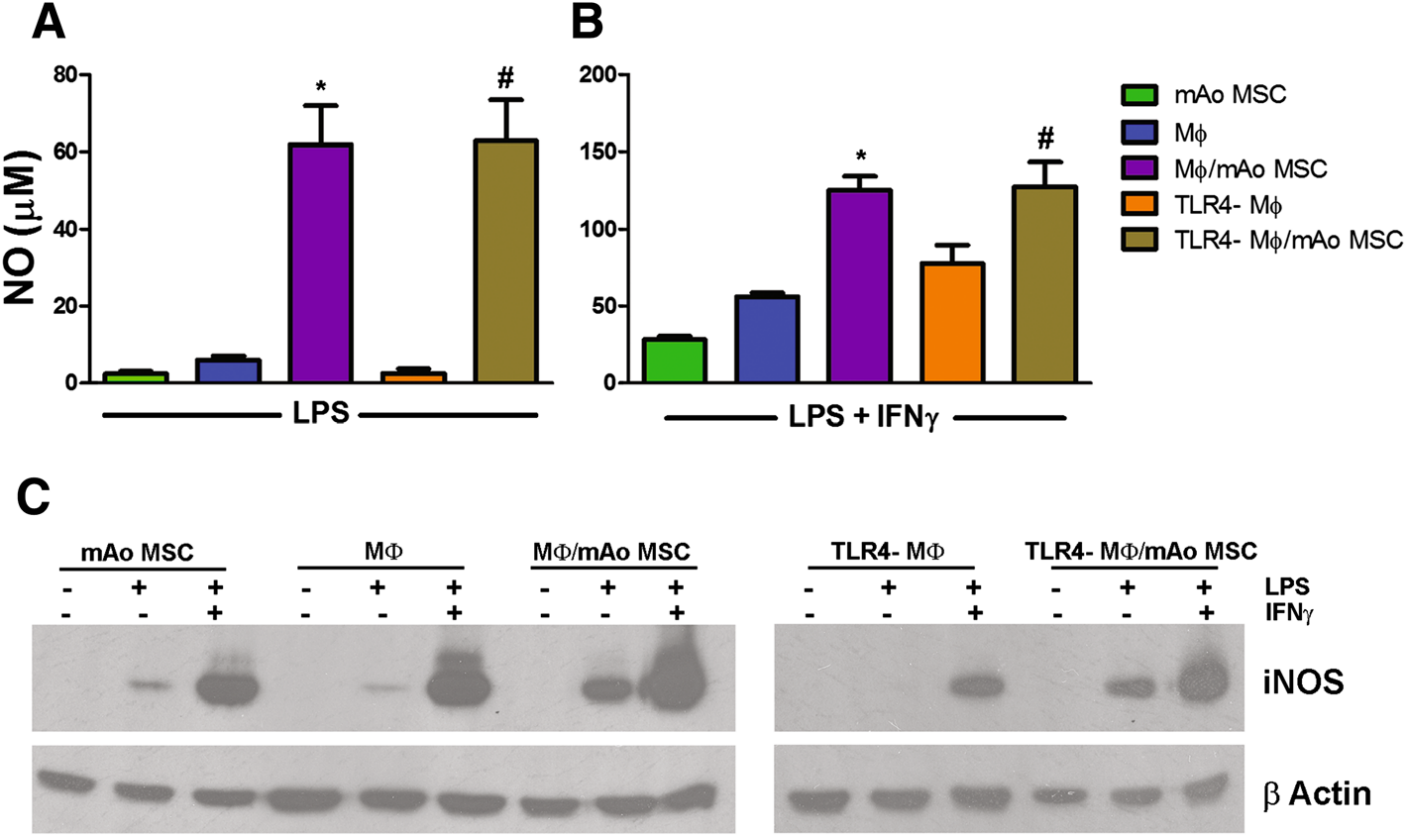
mAo cells in culture with MΦ cells contribute to local NO production in response to LPS and LPS/IFNγ. Nitrite production as a measure of NO in culture supernatants of mAo, MΦ, and splenic MΦ cells derived from TLR4-deficient mice (TLR4-MΦ) treated **(A)** with LPS (100 ng/mL) and **(B)** with LPS (100 ng/mL) + IFNγ (250 ng/mL) alone and in co-culture. Data are presented as mean ± standard error of the mean and are representative of three experiments each with n = 4. *Significantly different from MΦ cells alone; ^**#**^significantly different from TLR4-MΦ cells alone. **(C)** Representative Western blots from a separate experiment showing iNOS expression. Whole lysates were prepared from cultures, and 50 μg was loaded per lane. β-actin was used to control for protein loading. IFNγ, interferon-gamma; iNOS, inducible nitric oxide synthase; LPS, lipopolysaccharide; MΦ, bone marrow-derived macrophage; mAo, mouse aorta-derived mesenchymal progenitor; MSC, mesenchymal stem cell; NO, nitric oxide; TLR4, Toll-like receptor-4; TLR4-MΦ, Toll-like receptor-4-deficient macrophage.

We first examined NO production in response to inflammatory mediators by mAo and macrophage cells alone and in co-culture. NO production is a hallmark of the inflammatory M1 macrophage [16] but is also produced by some MSC populations to control T-cell proliferation and chemotaxis [17,18]. Untreated mAo cells, MΦ cells, and co-cultures produce undetectable levels of NO. When mAo cells were co-cultured with MΦ cells and exposed to LPS, they synergistically produced NO greater than 10-fold above either mAo or MΦ cells cultured alone. Co-cultures exposed to LPS and IFNγ in combination produced NO in an additive manner (Figure 1A,B).

To demonstrate the contribution of the mAo cells to the significantly elevated NO, we used co-cultures of mAo and TLR4-deficient splenic macrophage (TLR4-MΦ) cells which have a deficient inflammatory response to LPS [19]. As expected, TLR4-MΦ cells do not respond to LPS with an increase in NO. However, TLR4-MΦ/mAo cell co-cultures exposed to LPS demonstrate a significant increase in NO similar to the MΦ/mAo cell co-cultures (Figure 1A, B). Unexpectedly, TLR4-MΦ cells respond to LPS in the presence of IFNγ with significant NO production. In macrophage cells, Toll-like receptor-2 (TLR2) is upregulated by IFNγ and is capable of responding to LPS [20]. Therefore, it is likely that through upregulation and signaling through TLR2, the TLR4-MΦ cells produce NO in response to LPS/IFNγ in combination. Western blot demonstrated the upregulation of iNOS in association with elevated NO production, confirming activity of this enzyme as the source of NO (Figure 1C).

**Figure 2:**
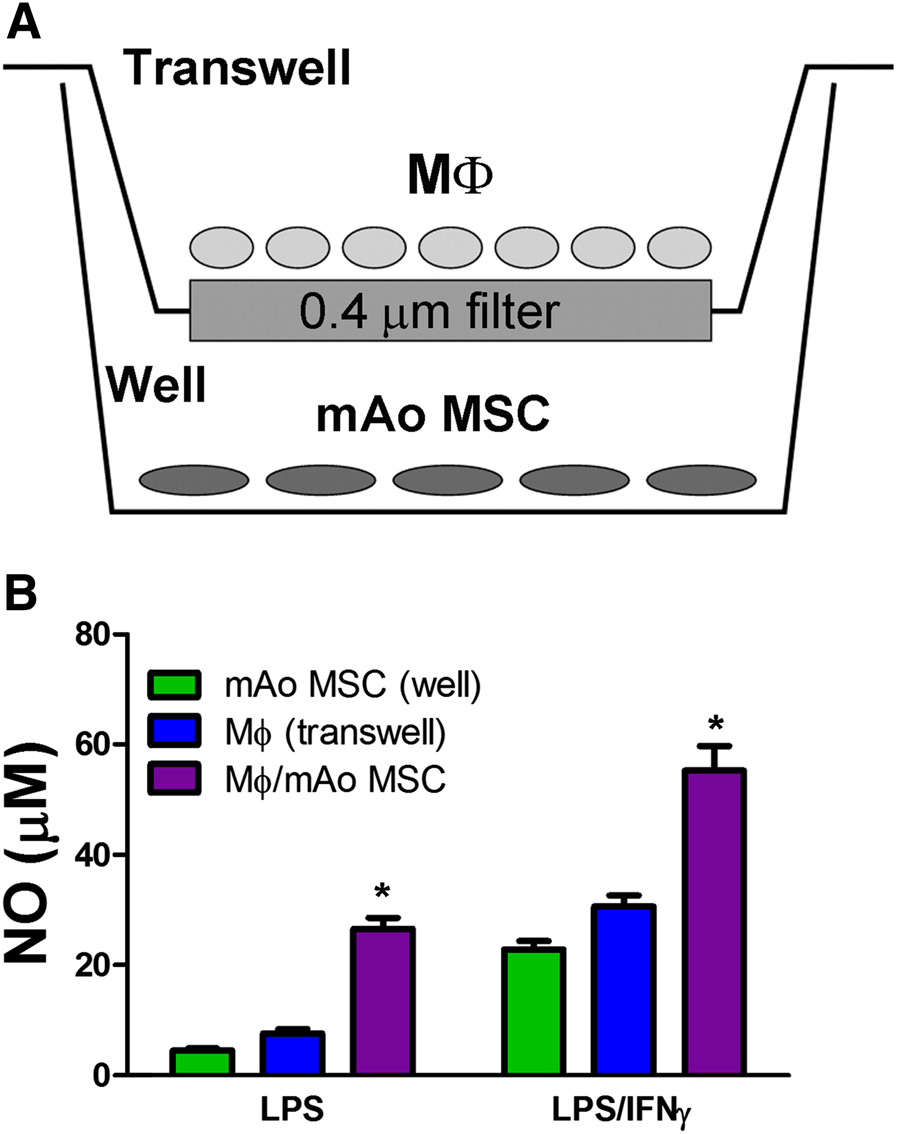
Cell-cell contact is required for the production of NO in MΦ/mAo cell co-cultures. MΦ and mAo MSCs were grown separated by a 0.4-μm filter **(A)** and activated with LPS (100 ng/mL) or with LPS (100 ng/mL) + IFNγ (250 ng/mL) for 24 hours. Supernatant of the mAo cells grown in the well and supernatant from the MΦ cells grown in the transwell were analyzed for NO content separately **(B)**. Data are presented as mean ± standard error of the mean and are representative of three experiments each with n = 4. *Significantly different from both transwell and well compartments. IFNγ, interferon-gamma; LPS, lipopolysaccharide; MΦ, bone marrow-derived macrophage; mAo, mouse aorta-derived mesenchymal progenitor; MSC, mesenchymal stem cell; NO, nitric oxide.

To determine whether cell-cell contact is required for the production of NO in MΦ/mAo cell co-cultures after activation, we used a transwell culture system (Figure 2A). mAo and MΦ cell populations were grown separated by a 0.4-μM filter which allows the passage of soluble factors between cell types but does not allow cell-cell contact. Both cell types were exposed to LPS or LPS and IFNγ, and the supernatants collected from the transwell compartment and the well compartment were analyzed for NO content separately with the expectation that NO concentrations in the compartments would be equivalent. Our results, however, reflect a gradient in NO diffusion, with NO concentrations greater near the MΦ cells in the transwell compartment. Regardless of the gradient, co-cultures grown in direct contact produce significantly greater NO than found in either compartment of the transwell culture system (Figure 2B).

### Interaction with mAo cell progenitors increases ox-LDL uptake by MΦ cells

**Figure 3:**
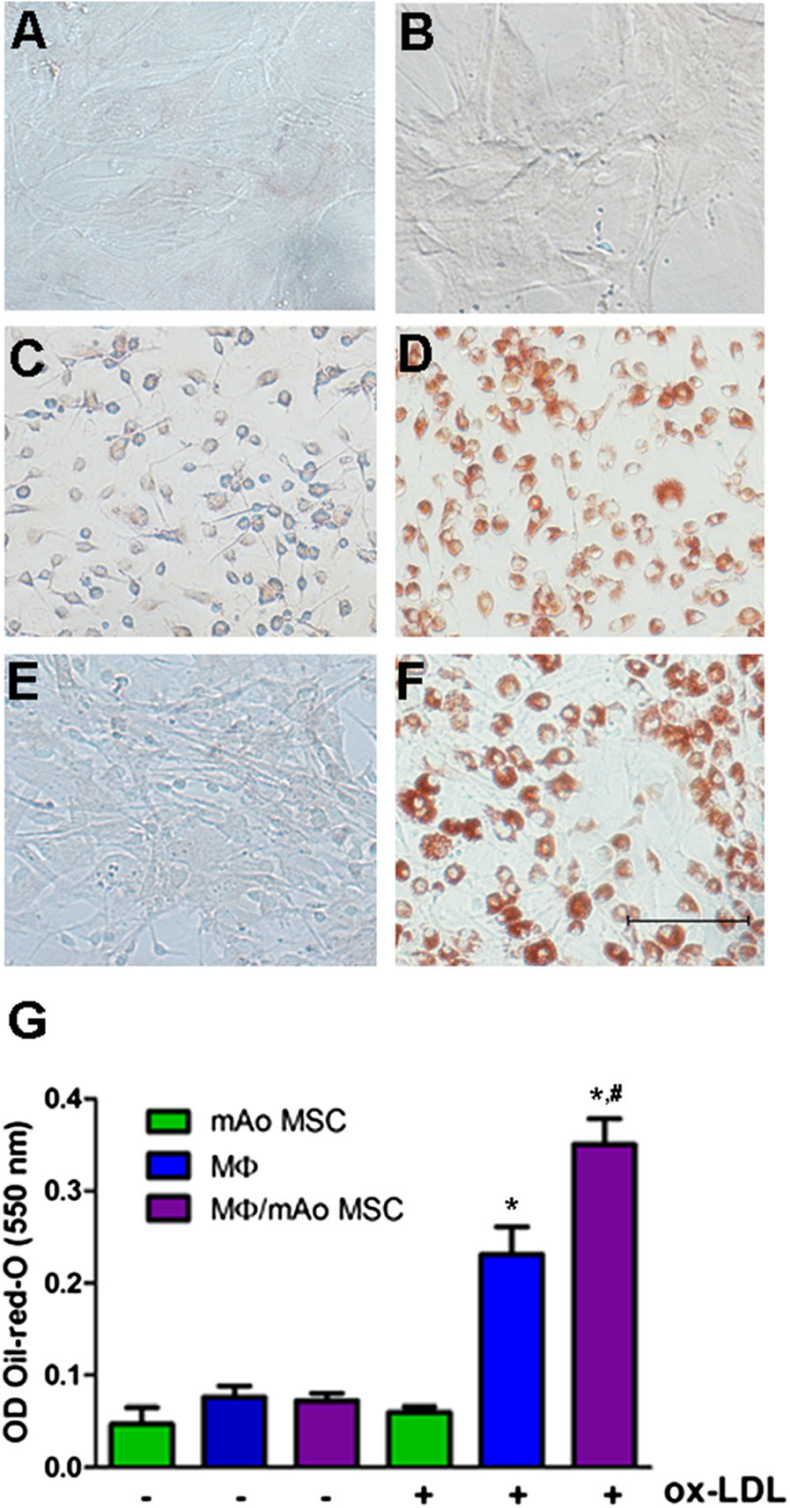
Interaction with mAo cells increases ox-LDL uptake by MΦ cells. Photomicrographs of Oil Red O stained cultures without (left frames) and with (right frames) ox-LDL (50 μg/mL) exposure for 24 hours. mAo cell cultures in **(A)** and **(B)**, MΦ cell cultures in **(C)** and **(D)**, and MΦ/mAo cell co-cultures in **(E)** and **(F)** are shown. Scale bar = 100 μM. **(G)** The spectrophotometric analysis of extracted Oil Red O stain. Data are presented as mean ± standard error of the mean and are representative of three experiments each with n = 4. *Significantly different from non-ox-LDL-treated counterpart; ^**#**^significantly different from MΦ cells alone under same conditions. MΦ, bone marrow-derived macrophage; mAo, mouse aorta-derived mesenchymal progenitor; OD, optical density; ox-LDL, oxidized low-density lipoprotein.

Vascular disease is associated with elevated levels of LDL and increased uptake of oxidized-LDL (ox-LDL) by MΦ cells [21]. To determine the effect that contact with the mAo cell progenitor has on MΦ cell uptake of ox-LDL, we exposed mAo and MΦ cells alone and in co-culture to ox-LDL. Ox-LDL uptake by MΦ cells was confirmed by Oil Red O staining, followed by photomicrograph and spectrophotometric analysis of extracted stain (Figure 3). No significant uptake of ox-LDL by mAo cells cultured alone was observed (Figure 3A,B), and a significant 1.5-fold increase in lipid staining was found in MΦ/mAo cell co-cultures compared with MΦ cells cultured alone (Figure 3C-G).

### MΦ and mAo cell progenitor interaction restores ox-LDL-suppressed local NO production in response to LPS and LPS/IFNγ

**Figure 4:**
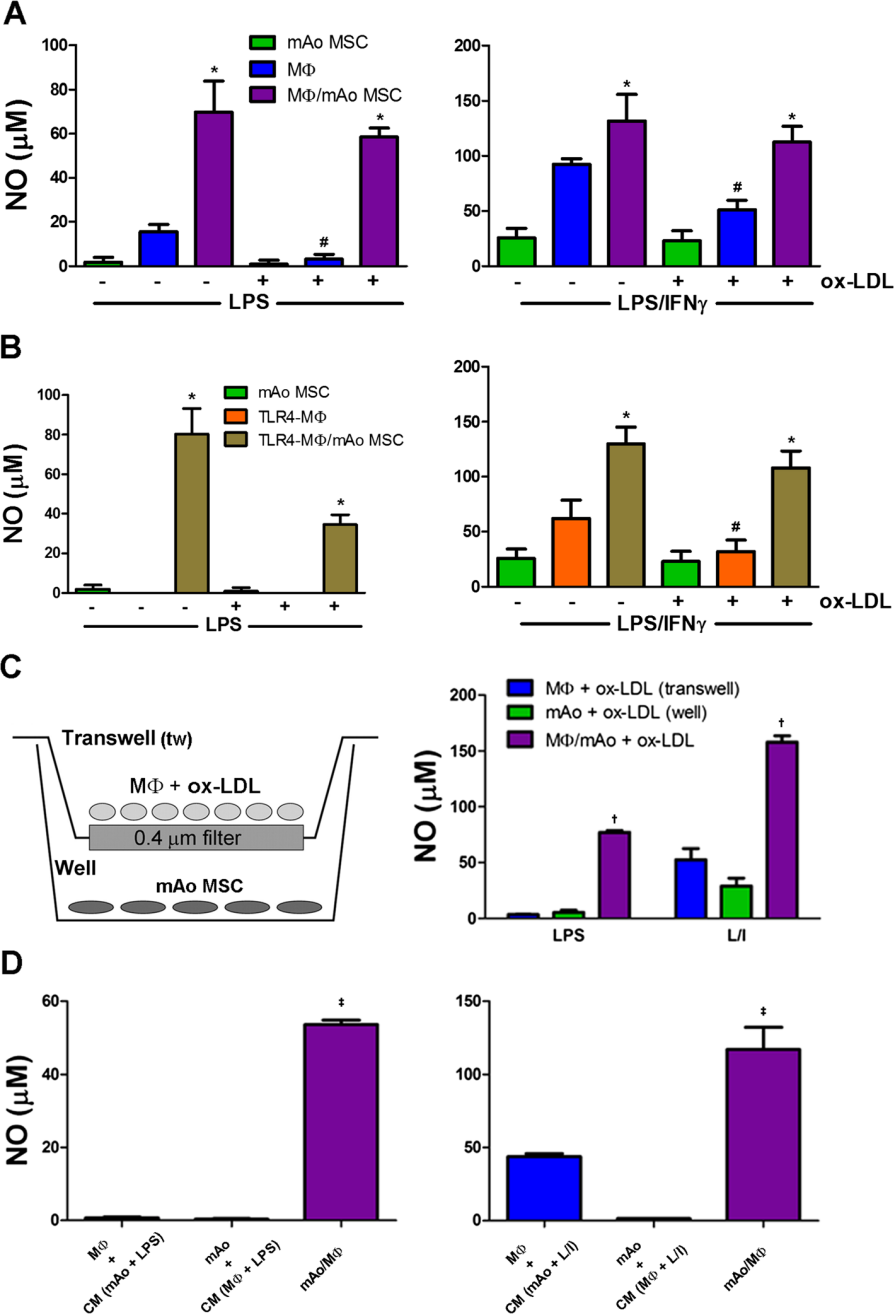
mAo and MΦ cell interaction restores ox-LDL-suppressed local NO production in response to LPS and LPS/IFNγ. Nitrite production is shown as a measure of NO in culture supernatants of MΦ cells **(A)** and TLR4-MΦ cells **(B)** alone and in co-culture with mAo cells after being treated with or without ox-LDL (50 μg/mL) for 24 hours and then activated with LPS (100 ng/mL) or with LPS (100 ng/mL) + IFNγ (250 ng/mL) for 24 hours. MΦ cells after being exposed to ox-LDL (50 μg/mL) for 24 hours were also cultured in transwells with mAo cells present in the well as depicted. Both MΦ cells in the transwell and mAo cells in the well were activated with LPS or LPS + IFNγ, and supernatants from the transwell and well were assayed for nitrate content separately **(C)**. Ox-LDL-treated mAo and MΦ cells were exposed to CM (collected after ox-LDL exposure and LPS and LPS + IFNγ (L/I) activation) from the opposing cell type. NO levels in the CM were subtracted from the supernatant values, and the net production is presented in **(D)**. Data are presented as mean ± standard error of the mean and are representative of three experiments each with n = 4. *Significantly different from MΦ or TLR4-MΦ cells alone under same conditions; ^**#**^significantly different from non-ox-LDL-treated counterpart; ^†^significantly different from well and transwell supernatant; ^‡^significantly different from cultures exposed to CM. CM, conditioned medium; IFNγ, interferon-gamma; LPS, lipopolysaccharide; MΦ, bone marrow-derived macrophage; mAo, mouse aorta-derived mesenchymal progenitor; MSC, mesenchymal stem cell; NO, nitric oxide; ox-LDL, oxidized low-density lipoprotein; TLR4-MΦ, Toll-like receptor-4-deficient macrophage.

Next, we sought to determine the influence of the mAo cells on MΦ cell production of NO after ox-LDL uptake. The uptake of ox-LDL by MΦ cells is associated with suppression of their NO production in response to inflammatory mediators [22] and our results confirm this finding. NO production is significantly reduced in MΦ cell cultures exposed to ox-LDL upon activation with LPS and LPS/IFNγ, approximately 5- and 1.8-fold, respectively (Figure 4A). However, LPS- and LPS/IFNγ-induced NO production in MΦ/mAo cell co-cultures returns to synergistic or additive levels found in the absence of ox-LDL (Figure 4A).

We used the TLR4-MΦ cells to determine the contribution of the mAo cells to NO production in ox-LDL-treated MΦ/mAo cell co-cultures. NO production was at similar levels in TLR4-MΦ/mAo and MΦ/mAo cell co-cultures after exposure to LPS, confirming the contribution of the mAo cells (Figure 4B). Again, the TLR4-MΦ cells respond to LPS/IFNγ with significant NO production, making it difficult to discern the contribution of the mAo cells.

To determine whether cell-cell contact is required for co-culture effects on NO production, mAo and MΦ cells exposed to ox-LDL were cultured together or separated by a filter with the use of transwell inserts (Figure 4C). NO production in ox-LDL-treated mAo and MΦ cell cultures separated by a filter is significantly lower, approximately 17-fold with LPS activation and approximately 3.8-fold with LPS/IFNγ activation, than co-cultures without a filter, indicating that cell-cell contact is required to recover production of NO (Figure 4C). We again found a gradient in NO concentration through the separate analysis of each compartment. Therefore, we used conditioned medium experiments to ensure that the cells were being exposed to the soluble factors produced by the opposing cell type after activation. We incubated mAo cell cultures with the conditioned medium of activated ox-LDL-treated MΦ cells and incubated ox-LDL-treated MΦ cells to the conditioned medium of activated mAo cells and measured the net NO production (Figure 4D). In line with the transwell cultures, NO produced by cultures exposed to conditioned medium was significantly less than when mAo and MΦ cells were cultured in direct contact.

### MΦ and mAo cell progenitor interaction restores ox-LDL-suppressed local LPS- and LPS/IFNγ-induced cytokine/chemokine production

**Figure 5:**
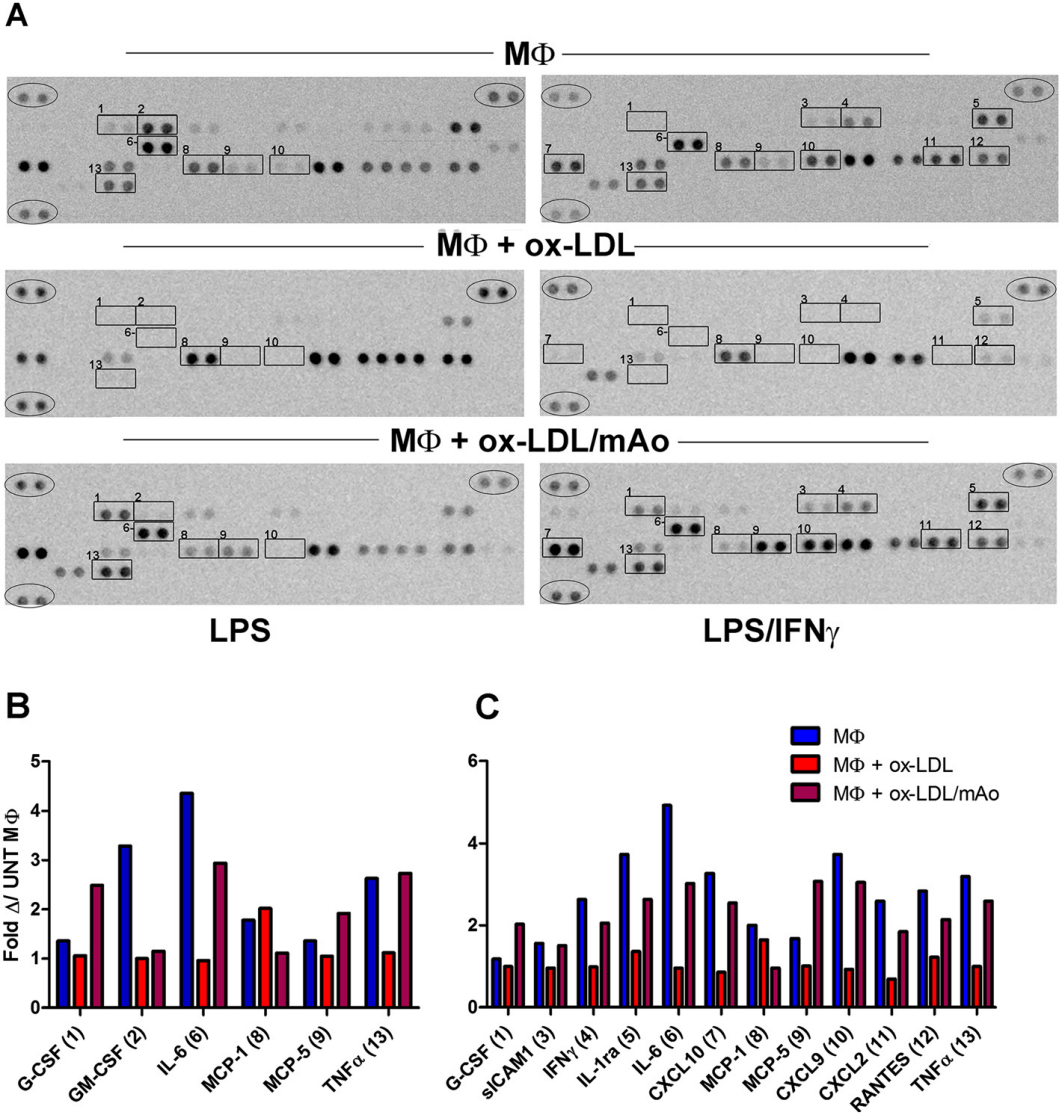
MΦ and mAo cell interaction restores ox-LDL-suppressed local LPS- and LPS/IFNγ-induced cytokine/chemokine production. Mouse cytokine Proteome Profiler immunoblots **(A)** and corresponding graphs of densitometry results **(B, C)** demonstrate changes in cytokine/chemokine levels in supernatants of MΦ cells, MΦ cells exposed to ox-LDL (50 μg/mL), and MΦ cells exposed to ox-LDL in co-culture with mAo cells. Cultures were activated with LPS (100 ng/mL) and LPS (100 ng/mL) + IFNγ (250 ng/mL) for 24 hours. Numbered boxes highlight cytokine/chemokines whose expression is changed with treatments in (A) and correspond with cytokine/chemokine numbers in (B) and (C). Circles highlight internal control spots used to normalize densitometric data. Graphs in (B) and (C) present the changes in spot density when compared with untreated MΦ cells (Fold Δ/UNT MΦ). The blot of the untreated MΦ cells can be viewed in Additional file 2: Figure S2 along with the overlay. The key for the spots can be found in Additional file 3. CXCL, chemokine (C-X-C motif) ligand; G-CSF, granulocyte colony-stimulating factor; GM-CSF, granulocyte/macrophage colony-stimulating factor; IFNγ, interferon-gamma; IL, interleukin; LPS, lipopolysaccharide; MΦ, bone marrow-derived macrophage; mAo, mouse aorta-derived mesenchymal progenitor; MCP, monocyte chemoattractant protein; ox-LDL, oxidized low-density lipoprotein; RANTES, regulated on activation, normal T-cell expressed and secreted; sICAM1, soluble intercellular adhesion molecule-1; TNFα, tumor necrosis factor-alpha.

Data are contradictory regarding inflammation-induced cytokine production in ox-LDL-laden MΦ cells. Some studies demonstrate a suppressed inflammatory response [23-25], whereas others claim that ox-LDL exposure enhances production of cytokines [26,27]. As an initial screen for co-culture-induced changes in the LPS- and LPS/IFNγ-responsive cytokine/chemokine secretion profile of lipid-laden MΦ cells, we used the mouse cytokine Proteome Profiler (R&D Systems). After uptake of ox-LDL, MΦ cell secretion of a number of growth factors, cytokines, and chemokines was reduced upon LPS and LPS/IFNγ exposure (Figure 5). MΦ/mAo cell interaction restored secretion of several chemokines involved in the summoning of the adaptive immunity—that is, CXCL2, CXCL9, CXCL10, and RANTES (regulated on activation, normal T-cell expressed and secreted). Although these and other changes in cytokine, chemokine, and growth factor secretions were observed—that is, co-cultures favored secretion of granulocyte colony-stimulating factor (G-CSF) over granulocyte/macrophage colony-stimulating factor (GM-CSF) as well as MCP-5 over MCP-1—we focused on the cytokines IL-6 and TNFα for this study. Production of these inflammatory cytokines is associated with vascular disease, and their significant secretion in response to LPS and LPS/IFNγ was restored by MΦ/mAo cell co-culture (Figure 5, Additional file 2: Figure S2 and Additional file 3).

### With exposure to ox-LDL, MΦ and mAo cell interaction restores LPS- and LPS/IFNγ-induced IL-6 secretion which is contributed by the mAo cell progenitor

**Figure 6:**
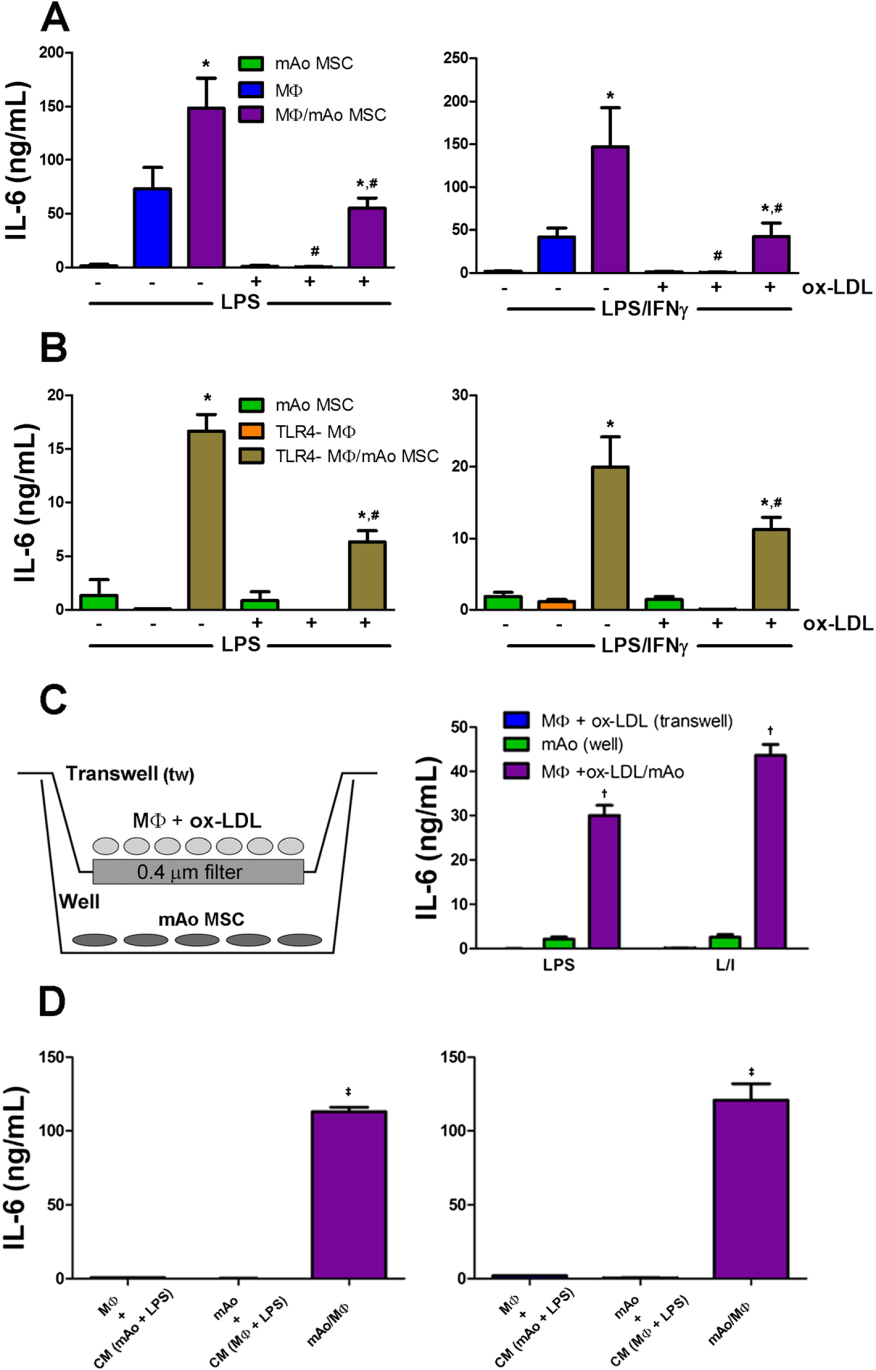
With exposure to ox-LDL, MΦ and mAo cell interaction restores LPS- and LPS/IFNγ-induced IL-6 secretion which is contributed by the mAo cells. Secreted IL-6 measured in culture supernatants of MΦ cells **(A)** and TLR4-MΦ cells **(B)** alone and in co-culture with mAo cells after being treated with or without ox-LDL (50 μg/mL) for 24 hours and then activated with LPS (100 ng/mL) or with LPS (100 ng/mL) + IFNγ (250 ng/mL) is shown. MΦ cells after being exposed to ox-LDL (50 μg/mL) for 24 hours were also cultured in transwells with mAo cells present in the well as depicted in **(C)**. Both MΦ cells in the transwell and mAo cells in the well were activated with LPS or LPS + IFNγ, and supernatants from the transwell and well compartments were assayed for IL-6 content separately (C). Ox-LDL-treated mAo and MΦ cells were exposed to CM (collected after ox-LDL exposure and LPS and LPS + IFNγ (L/I) activation) from the opposing cell type. IL-6 levels in the CM were subtracted from the supernatant values, and the net production is presented in **(D)**. Data are presented as mean ± standard error of the mean and are representative of three experiments each with n = 4. *Significantly different from MΦ or TLR4-MΦ cells alone under same conditions; ^**#**^significantly different from non-ox-LDL-treated counterpart; ^†^significantly different from well and transwell supernatant; ^‡^significantly different from cultures exposed to CM. CM, conditioned medium; IFNγ, interferon-gamma; IL-6, interleukin-6; LPS, lipopolysaccharide; MΦ, bone marrow-derived macrophage; mAo, mouse aorta-derived mesenchymal progenitor; MSC, mesenchymal stem cell; ox-LDL, oxidized low-density lipoprotein; TLR4-MΦ, Toll-like receptor-4-deficient macrophage.

Next, we confirmed the cytokine Proteome Profiler results for IL-6 and determined the contribution of the mAo cells to the restoration of local IL-6 secretion in ox-LDL-exposed cultures by using ELISA. In mAo cell cultures, LPS- and LPS/IFNγ-induced secretion of IL-6 is nominal compared with MΦ and MΦ/mAo cell cultures (Figure 6A). The interaction of mAo and MΦ cells results in a significant increase in production of IL-6 in response to LPS (approximately 2-fold increase) and LPS/IFNγ (approximately 3.5-fold increase) when compared with MΦ cells alone (Figure 6A). After uptake of ox-LDL, LPS- and LPS/IFNγ-induced IL-6 production is significantly suppressed in MΦ cell cultures by 167- and 61-fold, respectively. Under the same conditions, interaction of MΦ-mAo cells returns IL-6 secretion to levels observed in MΦ cell cultures not treated with ox-LDL (Figure 6A).

To determine whether the mAo cells directly contribute to the LPS- and LPS/IFNγ-induced IL-6 production in the presence and absence of ox-LDL, we used TLR4-MΦ and TLR4-MΦ/mAo cell cultures. As expected, activated TLR4-MΦ cells secrete IL-6 at levels significantly lower, more than 900-fold lower with LPS activation and 35-fold lower with LPS/IFNγ activation, than those of MΦ cell cultures not deficient in TLR4 (Figure 6B). However, in TLR4-MΦ/mAo cell cultures, IL-6 production is significantly increased and in the presence of ox-LDL remains at 37.5% and 56% of the levels measured in the absence of ox-LDL after LPS and LPS/IFNγ exposure, respectively.

To distinguish between a soluble factor and a direct cell contact-mediated mechanism, MΦ and mAo cells were separated by a transwell filter insert, treated with ox-LDL, and activated with LPS and LPS/IFNγ. Without direct contact, IL-6 production by MΦ and mAo cells is commensurate with the levels found when they are cultured alone (Figure 6C), indicating the involvement of a direct contact mechanism. Again, to ensure that the cells were being exposed to the soluble factors produced by the opposing activated cell type, we used conditioned medium experiments. mAo cell cultures incubated with the conditioned medium of activated ox-LDL-treated MΦ cells and ox-LDL-treated MΦ cells incubated with the conditioned medium of activated mAo cells produced IL-6 at significantly lower levels than when the cells are in direct contact, similar to the transwell cultures (Figure 6D).

### With exposure to ox-LDL, MΦ and mAo cell progenitor interaction restores significant LPS- and LPS/IFNγ-induced TNFα production by MΦ cells

**Figure 7:**
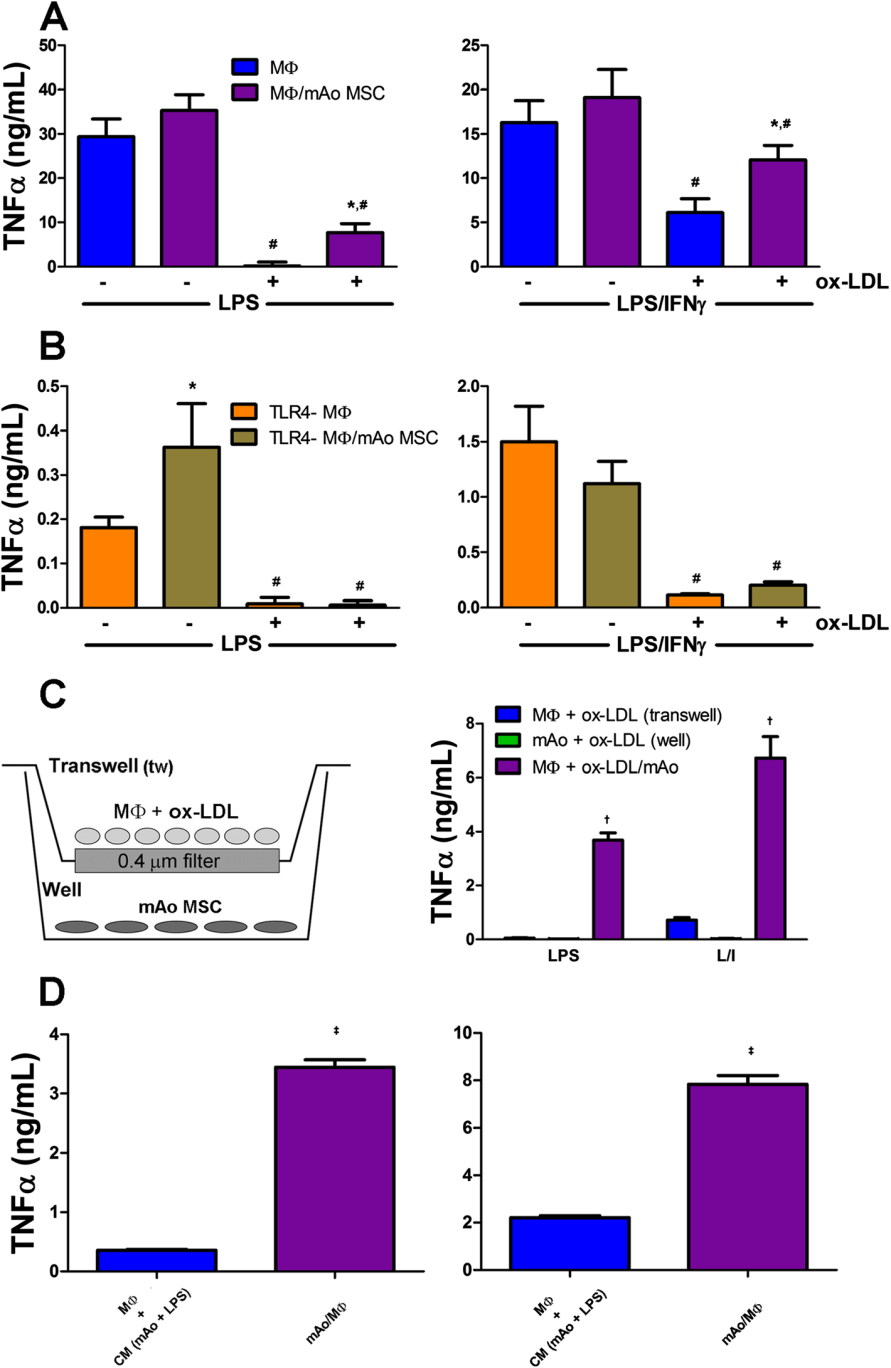
With exposure to ox-LDL, MΦ and mAo cell interaction restores significant LPS- and LPS/IFNγ-induced TNFα production by MΦ cells. Secreted TNFα measured in culture supernatants of MΦ cells **(A)** and TLR4-MΦ cells **(B)** alone and in co-culture with mAo cells after being treated with or without ox-LDL (50 μg/mL) for 24 hours and then activated with LPS (100 ng/mL) or with LPS (100 ng/mL) + IFNγ (250 ng/mL) is shown. MΦ cells after being exposed to ox-LDL (50 μg/mL) for 24 hours were also cultured in transwells with mAo cells present in the well as depicted in **(C)**. Both MΦ cells in the transwell and mAo cells in the well were activated with LPS or LPS + IFNγ, and supernatants from the transwell and well were assayed for TNFα content separately (C). Ox-LDL-treated mAo and MΦ cells were exposed to CM (collected after ox-LDL exposure and LPS and LPS + IFNγ (L/I) activation) from the opposing cell type. TNFα levels in the CM were subtracted from the supernatant values, and the net production is presented in **(D)**. Data are presented as mean ± standard error of the mean and are representative of three experiments each with n = 4. *Significantly different from MΦ or TLR4-MΦ cells alone under same conditions; ^#^significantly different from non-ox-LDL-treated counterpart; ^†^significantly different from well and transwell supernatant; ^‡^significantly different from cultures exposed to CM. CM, conditioned medium; IFNγ, interferon-gamma; LPS, lipopolysaccharide; MΦ, bone marrow-derived macrophage; mAo, mouse aorta-derived mesenchymal progenitor; MSC, mesenchymal stem cell; ox-LDL, oxidized low-density lipoprotein; TLR4-MΦ, Toll-like receptor-4-deficient macrophage; TNFα, tumor necrosis factor-alpha.

We also confirmed the cytokine Proteome Profiler results for TNFα and determined the contribution of the mAo cells to the restoration of local TNFα secretion in ox-LDL-exposed cultures by using ELISA. MSCs have been reported to be unable to produce TNFα [28,29], and we confirmed these findings in the mAo cell cultures. The TNFα protein was undetectable in the supernatant of these cells, and transcript expression was also out of detection range in mAo cells with or without treatment with inflammatory mediators (Additional file 4). After uptake of ox-LDL, LPS- and LPS/IFNγ-induced TNFα production is significantly suppressed in MΦ cell cultures by 155- and 4.6-fold, respectively. Under the same conditions, interaction of MΦ and mAo cells returns TNFα to 21.7% and 63% of levels observed in non-ox-LDL-treated MΦ/mAo cell co-cultures after treatment with LPS or LPS/IFNγ, respectively (Figure 7A).

To confirm the expectation that mAo cells do not directly contribute to the LPS- and LPS/IFNγ-induced TNFα production in the presence and absence of ox-LDL, we used TLR4-MΦ and TLR4-MΦ/mAo cell cultures. As expected, activated TLR4-MΦ cells secrete TNFα at levels significantly lower, more than 150-fold lower with LPS and more than 10-fold lower with LPS/IFNγ activation, than those of MΦ cell cultures not deficient in TLR4 (Figure 7B). In TLR4-MΦ/mAo cell cultures under conditions of suppressed MΦ cell response (that is, in the presence of ox-LDL and TLR4 deficiency), TNFα production, unlike IL-6 production, is not significantly increased above TLR4-MΦ cells alone.

The transwell system was again used to determine whether the MΦ-mAo cell interaction effect on TNFα secretion is dependent upon a soluble factor or a direct cell contact-mediated mechanism. Without direct contact with mAo cells, TNFα production by MΦ cells is commensurate with the level found when they are cultured alone (Figure 7C), indicating the involvement of a direct contact mechanism. These data were also confirmed with conditioned medium studies (Figure 7D).

## Discussion

It is well known that mesenchymal progenitors can suppress immune responses [1]. However, these cells can also support inflammatory responses and therefore have been termed ‘sensors and switchers’ of inflammation [17,18]. In fact, a recent paradigm suggests a polarization similar to that of leukocyte activation in which both pro-inflammatory and immunosuppressive mesenchymal progenitor phenotypes can develop dependent upon the environment [12,28]. According to this paradigm, mAo cells, when in direct contact with MΦ cells and exposed to ox-LDL and inflammatory mediators, are pro-inflammatory. mAo cells contribute to local production of both NO and IL-6 and enhance ox-LDL uptake by macrophage cells. MΦ cells exposed to ox-LDL experience suppressed secretion of NO, IL-6, and TNFα in response to inflammatory mediators, and the interaction with mAo cells restored significant secretion of these inflammatory markers. mAo cells contribute to the restoration of the inflammatory response both directly through production of NO and IL-6 and indirectly by promoting TNFα secretion by the MΦ cells. Both transwell and conditioned medium experiments suggest a direct contact mechanism for this restoration.

It should be noted that data regarding the inflammatory response of lipid-laden MΦ cells are conflicting. Older studies using RAW 264.7 MΦ, murine bone marrow-derived, and peritoneal MΦ cells demonstrate a suppression of inflammatory cytokine secretion after ox-LDL exposure [21-25]. More recently, others have demonstrated that ox-LDL loading enhances the inflammatory response of MΦ cells [26,27]. The study by Groeneweg and colleagues [26], like our study, used bone marrow-derived MΦ cells from C57BL/6 mice which were propagated in medium supplemented with L-929 fibroblast conditioned medium. They found that priming MΦ cells with ox-LDL increased their production of IL-6 and TNFα in response to LPS. Yet in our hands, these cells experience a significantly suppressed response to LPS and LPS/IFNγ after ox-LDL loading. Perhaps this discrepancy may be explained by the conditions in which the MΦ cell cultures were activated with the inflammatory mediators. Our cultures were exposed to LPS and LPS/IFNγ in a serum-free medium supplemented with 0.2% BSA, but in the study by Groeneweg and colleagues, the LPS was added to medium supplemented with FBS. The response to inflammatory cues by MΦ cells in a lipid-laden environment needs further exploration.

The criteria for applying the designation of multipotent mesenchymal stromal cells also known as MSCs have been an area of contention in mesenchymal progenitor research. In 2006, the Mesenchymal and Tissue Stem Cell Committee of the International Society for Cellular Therapy published a position paper outlining minimum criteria for the MSC designation in human cell populations [30]. For mouse cells, however, no clear criteria have emerged, and there are many differences in MSC surface antigen expression between mouse and human cells [31]. There is also a great variation in reported MSC surface antigen expression, and many new potential identifying antigens are continually emerging [32]. Therefore, the concept of MSCs has become increasingly ambiguous. The mAo cells used in this study have the potential to differentiate into osteoblast, adipocyte, and chondrogenic lineages and express several surface antigens associated with mouse MSCs. However, the ‘stemness’ of these cells has not been completely characterized; hence, we have used the term mAo cell progenitor.

Many recent studies explore the relationship between heterogeneity in surface antigen expression and heterogeneity in functional properties among MSCs. For example, MSCs isolated from adipose (AdMSCs) are heterogenic in CD105 (endoglin) expression, and CD105^+^ AdMSCs are functionally distinct from those that are CD105^−^ [33]. In line with this study, surface antigen expression in MSCs and more differentiated mesenchymal cell populations also appears closely associated with immunoregulatory function. mAo cell progenitors and vascular smooth muscle cells in contact with monocytes are pro-inflammatory and are CD105^+^/CD73^−^ [34-36], whereas bone marrow-derived MSCs (BMMSCs) and AdMSCs are CD105^−^/CD73^+^ and promote the differentiation of the anti-inflammatory macrophage [33,37]. CD105^+^ AdMSCs have a reduced capacity to regulate T-cell proliferation [33], and CD73 or ecto-5′-nucleotidase catalyzes the conversion of 5′-AMP to adenosine and plays an important role in suppressing inflammation [38]. Expression of CD105 and the lack of CD73 in mAo cells could be why they, unlike BMMSCs and AdMSCs, do not suppress but support the inflammatory response of macrophage cells. CD105 expression is elevated in atherosclerotic plaque [39], and inactivation of CD73 promotes atherogenesis in apolipoprotein E-deficient mice [38], correlating our *in vitro* data to data from *in vivo* studies.

We used the TLR-4-deficient splenic macrophage (TLR4-MΦ) cells, which are unresponsive to LPS, to determine the contribution of the mAo cell progenitor to the inflammatory response. However, in the presence of IFNγ, the TLR4-MΦ cells were capable of responding to LPS and therefore the precise contribution from the mAo cell progenitor in the presence of IFNγ remains to be determined. In macrophage cells, TLR2 is upregulated by IFNγ and can be activated by LPS [20]. Therefore, it is likely that through upregulation and signaling through TLR2, the TLR4-MΦ cells produce inflammatory mediators in response to LPS/IFNγ in combination. However, transwell and conditioned medium experiments confirm that cell contact between the mAo cell progenitors and the macrophage cells is required for the inflammatory response in the presence of LPS/IFNγ.

In the indirect co-culture studies, analysis of supernatant from the transwell and the well compartments separately revealed the presence of a diffusion gradient between the compartments after 24 hours of incubation. To ensure that the cells were exposed to secreted soluble factors at the concentrations that would be present if the cells were in immediate proximity, we used conditioned medium experiments. Although our findings were confirmed, the presence of a gradient identifies a potential limitation to using the transwell for indirect co-culture studies.

It is clear that the MΦ-mAo cell interaction contributes to the inflammatory response; however, the roles of NO, IL-6, and TNFα in the progression of vascular disease remain unclear. Elevated NO levels can contribute to local tissue damage and macrophage apoptosis [40,41], but NO can also suppress LDL oxidation [42-45]. Exogenous IL-6 treatment in apolipoprotein E-deficient (ApoE^−/−^) mice results in an enhancement of lesions [46], but a lifetime of IL-6 deficiency in this same animal model leads to a similar result [47]. IL-6 also protects human macrophages from cellular cholesterol accumulation through enhancing ATP-binding cassette transporter 1 (ABCA1)-mediated cholesterol efflux [48]. Exposure to TNFα for 24 hours reduces scavenger receptor expression in macrophage and subsequent ox-LDL uptake [49,50]. On the other hand, TNFα exposure at 1 to 10 ng/mL is associated with reductions in macrophage efferocytosis [51]. Therefore, synergism in the production of IL-6, TNFα, and NO in the early stages of vascular disease could be acting in concert against further atherosclerotic progression by reducing local LDL oxidation, reducing ox-LDL uptake through suppression of scavenger receptors, and enhancing cholesterol efflux. However, in later stages, this continued synergism could lead to increased macrophage apoptosis, decreased efferocytosis, and subsequent plaque instability.

In lipid-laden MΦ cell cultures, the secretion of several chemokines was suppressed in response to inflammatory mediators but was restored upon co-culture with mAo cells. These (C-X-C motif ligand) chemokines—CXCL2, CXCL9, CXCL10, and CCL5 (RANTES)—play roles in leukocyte recruitment and interfacing the innate with the inflammatory adaptive immunity [9]. It is also interesting to note the switch from MCP-1 secretion in activated macrophage cultures to MCP-5 secretion in activated co-cultures of MΦ and mAo cells. MCP-5 is the mouse orthologue to MCP-1 in humans and is expressed in ovalbumin-treated macrophage and airway smooth muscle cells [52]. Although MCP-5 is chemotactic for monocytes, lymphocytes, and eosinophils, among lymphocytes it preferentially attracts the B1 B-lymphocyte *in vitro* [52]. The B1 B-lymphocyte has been characterized as anti-atherogenic [53]. The role of MCP-5 in vascular disease warrants further investigation.

## Conclusions

The interaction between macrophage cells and mAo cell progenitors under inflammatory conditions is synergistic in the secretion of the NO, IL-6, and TNFα inflammatory markers. After uptake of ox-LDL, MΦ cell interaction with mAo cells re-establishes the inflammatory response as well as the interface between the innate and adaptive immunities. Early during vascular disease progression, this may be beneficial, but continued support of inflammatory activity in later stages of disease could lead to irreparable damage. The vascular mesenchymal progenitor and its contact with lipid-laden macrophage represent an additional target for therapeutics in chronic vascular inflammation.

## Additional files

Additional file 1: Figure S1. Characterization of the aorta-derived progenitor (mAo) cell line and bone marrow-derived macrophage (MΦ). Flow cytometry results demonstrate expression of mesenchymal stem cell (MSC)-associated surface antigens in the mAo cell cultures **(A)** and the expression of the MOMA-2 monocyte/macrophage-associated intracellular antigen by the MΦ cultures **(B)**. **(C)** Photomicrographs of MΦ cultures stained with non-specific esterase (NSE) in the presence of fluoride and counterstained with hematoxylin to demonstrate the nuclei. mAo cells were stained with Alcian Blue/von Kossa (AB/VK) stain after culture in chondrogenic conditions. Additional file 2: Figure S2. Proteome Profiler cytokine/chemokine blot of untreated MΦ culture which was used as a baseline to analyze data presented in Figure 5. Densitometric data from mouse cytokine Proteome Profiler immunoblots shown in Figure 5 were compared with densitometric analysis of the untreated MΦ blot shown here **(A)**. **(B)** The overlay of coordinates. The key is found in Additional file 3. MΦ, bone marrow-derived macrophage. Additional file 3:
**The key for the Proteome Profiler coordinates found in Additional file** **2** **: Figure S2.**
Additional file 4:
**Secreted tumor necrosis factor-alpha (TNFα) protein and TNFα transcript are below level of detection in mAo cell cultures.** An Excel file shows standard curves and assay results from enzyme-linked immunosorbent assay (ELISA) and real-time polymerase chain reaction (PCR) for TNFα expression in mAo cell cultures. mAo, mouse aorta-derived mesenchymal progenitor.

## Abbreviations

AdMSC: mesenchymal stem cell isolated from adipose
ATCC: American Type Tissue Collection
BMMSC: bone marrow-derived mesenchymal stem cell
BSA: bovine serum albumin
CXCL: chemokine (C-X-C motif) ligand
DMEM: Dulbecco’s modified Eagle’s medium
ELISA: enzyme-linked immunosorbent assay
FBS: fetal bovine serum
FITC: fluorescein isothiocyanate
IFNγ: interferon-gamma
IL: interleukin
iNOS: inducible nitric oxide synthase
LDL: low-density lipoprotein
LPS: lipopolysaccharide
MΦ: bone marrow-derived macrophage
M1: traditionally activated macrophage
M2: alternatively activated macrophage
mAo: mouse aorta-derived mesenchymal progenitor
MCP: monocyte chemoattractant protein
M-CSF: macrophage colony-stimulating factor
MSC: mesenchymal stem cell
NO: nitric oxide
ox-LDL: oxidized low-density lipoprotein
PBS: phosphate-buffered saline
PE: phycoerythrin
RANTES: regulated on activation, normal T-cell expressed and secreted
TBST: Tris-buffered saline with 0.1% tween-20
TLR: Toll-like receptor
TLR4-MΦ: Toll-like receptor-4-deficient macrophage
TNFα: tumor necrosis factor-alpha

## References

[1] Prockop DJ, Oh JY (2012). Mesenchymal stem/stromal cells (MSCs): role as guardians of inflammation. Mol Ther.

[2] Zhang L, Xu Q (2014). Stem/Progenitor cells in vascular regeneration. Arterioscler Thromb Vasc Biol.

[3] Abumaree MH, Al Jumah MA, Kalionis B, Jawdat D, Al Khaldi A, Abomaray FM (2013). Human placental mesenchymal stem cells (pMSCs) play a role as immune suppressive cells by shifting macrophage differentiation from inflammatory M1 to anti-inflammatory M2 macrophages. Stem Cell Rev.

[4] Cho DI, Kim MR, Jeong HY, Jeong HC, Jeong MH, Yoon SH (2014). Mesenchymal stem cells reciprocally regulate the M1/M2 balance in mouse bone marrow-derived macrophages. Exp Mol Med.

[5] Maggini J, Mirkin G, Bognanni I, Holmberg J, Piazzon IM, Nepomnaschy I (2010). Mouse bone marrow-derived mesenchymal stromal cells turn activated macrophages into a regulatory-like profile. PLoS One.

[6] da Silva ML, Chagastelles PC, Nardi NB (2006). Mesenchymal stem cells reside in virtually all post-natal organs and tissues. J Cell Sci.

[7] Psaltis PJ, Harbuzariu A, Delacroix S, Holroyd EW, Simari RD (2011). Resident vascular progenitor cells–diverse origins, phenotype, and function. J Cardiovasc Transl Res.

[8] Tabas I (2010). Macrophage death and defective inflammation resolution in atherosclerosis. Nat Rev Immunol.

[9] Mantovani A, Garlanda C, Locati M (2009). Macrophage diversity and polarization in atherosclerosis: a question of balance. Arterioscler Thromb Vasc Biol.

[10] Leitinger N, Schulman IG (2013). Phenotypic polarization of macrophages in atherosclerosis. Arterioscler Thromb Vasc Biol.

[11] Ley K, Miller YI, Hedrick CC (2011). Monocyte and macrophage dynamics during atherogenesis. Arterioscler Thromb Vasc Biol.

[12] Waterman RS, Tomchuck SL, Henkle SL, Betancourt AM (2010). A new mesenchymal stem cell (MSC) paradigm: polarization into a pro-inflammatory MSC1 or an Immunosuppressive MSC2 phenotype. PLoS One.

[13] Anton K, Banerjee D, Glod J (2012). Macrophage-associated mesenchymal stem cells assume an activated, migratory, pro-inflammatory phenotype with increased IL-6 and CXCL10 secretion. PLoS One.

[14] Evans JF, Fernando A, Ragolia L (2012). Functional melanocortin-2 receptors are expressed by mouse aorta-derived mesenchymal progenitor cells. Mol Cell Endocrinol.

[15] Yeh JK, Evans JF, Chen MM, Aloia JF (1999). Effect of hypophysectomy on the proliferation and differentiation of rat bone marrow stromal cells. Am J Physiol.

[16] Sica A, Mantovani A (2012). Macrophage plasticity and polarization: in vivo veritas. J Clin Invest.

[17] Bernardo ME, Fibbe WE (2013). Mesenchymal stromal cells: sensors and switchers of inflammation. Cell Stem Cell.

[18] Li W, Ren G, Huang Y, Su J, Han Y, Li J (2012). Mesenchymal stem cells: a double-edged sword in regulating immune responses. Cell Death Differ.

[19] Palsson-McDermott EM, O’Neill LA (2004). Signal transduction by the lipopolysaccharide receptor, Toll-like receptor-4. Immunology.

[20] Matsuguchi T, Musikacharoen T, Ogawa T, Yoshikai Y (2000). Gene expressions of Toll-like receptor 2, but not Toll-like receptor 4, is induced by LPS and inflammatory cytokines in mouse macrophages. J Immunol.

[21] Min KJ, Cho KH, Kwon TK (2012). The effect of oxidized low density lipoprotein (oxLDL)-induced heme oxygenase-1 on LPS-induced inflammation in RAW 264.7 macrophage cells. Cell Signal.

[22] Matthys KE, Jorens PG, Marescau B, Rosseneu M, Bult H, Herman AG (1994). Oxidized lipoproteins suppress nitric oxide synthase in macrophages: study of glucocorticoid receptor involvement. Mediators Inflamm.

[23] Chung SW, Kang BY, Kim SH, Pak YK, Cho D, Trinchieri G (2000). Oxidized low density lipoprotein inhibits interleukin-12 production in lipopolysaccharide-activated mouse macrophages via direct interactions between peroxisome proliferator-activated receptor-gamma and nuclear factor-kappa B. J Biol Chem.

[24] Fong LG, Fong TA, Cooper AD (1991). Inhibition of lipopolysaccharide-induced interleukin-1 beta mRNA expression in mouse macrophages by oxidized low density lipoprotein. J Lipid Res.

[25] Hamilton TA, Ma GP, Chisolm GM (1990). Oxidized low density lipoprotein suppresses the expression of tumor necrosis factor-alpha mRNA in stimulated murine peritoneal macrophages. J Immunol.

[26] Groeneweg M, Kanters E, Vergouwe MN, Duerink H, Kraal G, Hofker MH (2006). Lipopolysaccharide-induced gene expression in murine macrophages is enhanced by prior exposure to oxLDL. J Lipid Res.

[27] van Tits LJ, Stienstra R, van Lent PL, Netea MG, Joosten LA, Stalenhoef AF (2011). Oxidized LDL enhances pro-inflammatory responses of alternatively activated M2 macrophages: a crucial role for Kruppel-like factor 2. Atherosclerosis.

[28] Romieu-Mourez R, Francois M, Boivin MN, Bouchentouf M, Spaner DE, Galipeau J (2009). Cytokine modulation of TLR expression and activation in mesenchymal stromal cells leads to a proinflammatory phenotype. J Immunol.

[29] van den Berk LC, Jansen BJ, Siebers-Vermeulen KG, Roelofs H, Figdor CG, Adema GJ (2010). Mesenchymal stem cells respond to TNF but do not produce TNF. J Leukoc Biol.

[30] Dominici M, Le Blanc K, Mueller I, Slaper-Cortenbach I, Marini F, Krause D (2006). Minimal criteria for defining multipotent mesenchymal stromal cells, The International Society for Cellular Therapy position statement. Cytotherapy.

[31] Kolf CM, Cho E, Tuan RS (2007). Mesenchymal stromal cells. Biology of adult mesenchymal stem cells: regulation of niche, self-renewal and differentiation. Arthritis Res Ther.

[32] Lv FJ, Tuan RS, Cheung KM, Leung VY (2014). Concise review: the surface markers and identity of human mesenchymal stem cells. Stem Cells.

[33] Anderson P, Carrillo-Galvez AB, Garcia-Perez A, Cobo M, Martin F (2013). CD105 (endoglin)-negative murine mesenchymal stromal cells define a new multipotent subpopulation with distinct differentiation and immunomodulatory capacities. PLoS One.

[34] Chen L, Frister A, Wang S, Ludwig A, Behr H, Pippig S (2009). Interaction of vascular smooth muscle cells and monocytes by soluble factors synergistically enhances IL-6 and MCP-1 production. Am J Physiol Heart Circ Physiol.

[35] Butoi ED, Gan AM, Manduteanu I, Stan D, Calin M, Pirvulescu M (1813). Cross talk between smooth muscle cells and monocytes/activated monocytes via CX3CL1/CX3CR1 axis augments expression of pro-atherogenic molecules. Biochim Biophys Acta.

[36] Ikeda U, Maeda Y, Funayama H, Hojo Y, Ikeda M, Minota S (1998). Monocyte-vascular smooth muscle cell interaction enhances nitric oxide production. Cardiovasc Res.

[37] Tigges U, Komatsu M, Stallcup WB (2012). Adventitial pericyte progenitor/mesenchymal stem cells participate in the restenotic response to arterial injury. J Vasc Res.

[38] Buchheiser A, Ebner A, Burghoff S, Ding Z, Romio M, Viethen C (2011). Inactivation of CD73 promotes atherogenesis in apolipoprotein E-deficient mice. Cardiovasc Res.

[39] Bot PT, Hoefer IE, Sluijter JP, van Vliet P, Smits AM, Lebrin F (2009). Increased expression of the transforming growth factor-beta signaling pathway, endoglin, and early growth response-1 in stable plaques. Stroke.

[40] Martinet W, Croons V, Timmermans JP, Herman AG, De Meyer GR (2007). Nitric oxide selectively depletes macrophages in atherosclerotic plaques via induction of endoplasmic reticulum stress. Br J Pharmacol.

[41] Wang BY, Ho HK, Lin PS, Schwarzacher SP, Pollman MJ, Gibbons GH (1999). Regression of atherosclerosis: role of nitric oxide and apoptosis. Circulation.

[42] Ahmed A, Fujisawa T, Niu XL, Ahmad S, Al-Ani B, Chudasama K (2009). Angiopoietin-2 confers Atheroprotection in apoE-/- mice by inhibiting LDL oxidation via nitric oxide. Circ Res.

[43] Niu XL, Chen Y, Shoyama Y, Ishiwata K, Obama R, Nakazawa H (2002). Inducible nitric oxide synthase knockout mouse and low-density lipoprotein oxidation. Methods Enzymol.

[44] Rubbo H, O’Donnell V (2005). Nitric oxide, peroxynitrite and lipoxygenase in atherogenesis: mechanistic insights. Toxicology.

[45] Yates MT, Lambert LE, Whitten JP, McDonald I, Mano M, Ku G (1992). A protective role for nitric oxide in the oxidative modification of low density lipoproteins by mouse macrophages. FEBS Lett.

[46] Huber SA, Sakkinen P, Conze D, Hardin N, Tracy R (1999). Interleukin-6 exacerbates early atherosclerosis in mice. Arterioscler Thromb Vasc Biol.

[47] Schieffer B, Selle T, Hilfiker A, Hilfiker-Kleiner D, Grote K, Tietge UJ (2004). Impact of interleukin-6 on plaque development and morphology in experimental atherosclerosis. Circulation.

[48] Frisdal E, Lesnik P, Olivier M, Robillard P, Chapman MJ, Huby T (2011). Interleukin-6 protects human macrophages from cellular cholesterol accumulation and attenuates the proinflammatory response. J Biol Chem.

[49] Hsu HY, Nicholson AC, Hajjar DP (1996). Inhibition of macrophage scavenger receptor activity by tumor necrosis factor-alpha is transcriptionally and post-transcriptionally regulated. J Biol Chem.

[50] Hsu HY, Twu YC (2000). Tumor necrosis factor-alpha -mediated protein kinases in regulation of scavenger receptor and foam cell formation on macrophage. J Biol Chem.

[51] Michlewska S, Dransfield I, Megson IL, Rossi AG (2009). Macrophage phagocytosis of apoptotic neutrophils is critically regulated by the opposing actions of pro-inflammatory and anti-inflammatory agents: key role for TNF-alpha. FASEB J.

[52] Jia GQ, Gonzalo JA, Lloyd C, Kremer L, Lu L, Martinez AC (1996). Distinct expression and function of the novel mouse chemokine monocyte chemotactic protein-5 in lung allergic inflammation. J Exp Med.

[53] Lichtman AH, Binder CJ, Tsimikas S, Witztum JL (2013). Adaptive immunity in atherogenesis: new insights and therapeutic approaches. J Clin Invest.

