# Supplementary figures and images for: Mouse aorta-derived mesenchymal progenitor cells contribute to and enhance the immune response of macrophage cells under inflammatory conditions
Source: Stem Cell Res Ther. 2015 Apr 14;6(1):56. doi: 10.1186/s13287-015-0071-8 (PMC4414009; doi:10.1186/s13287-015-0071-8)

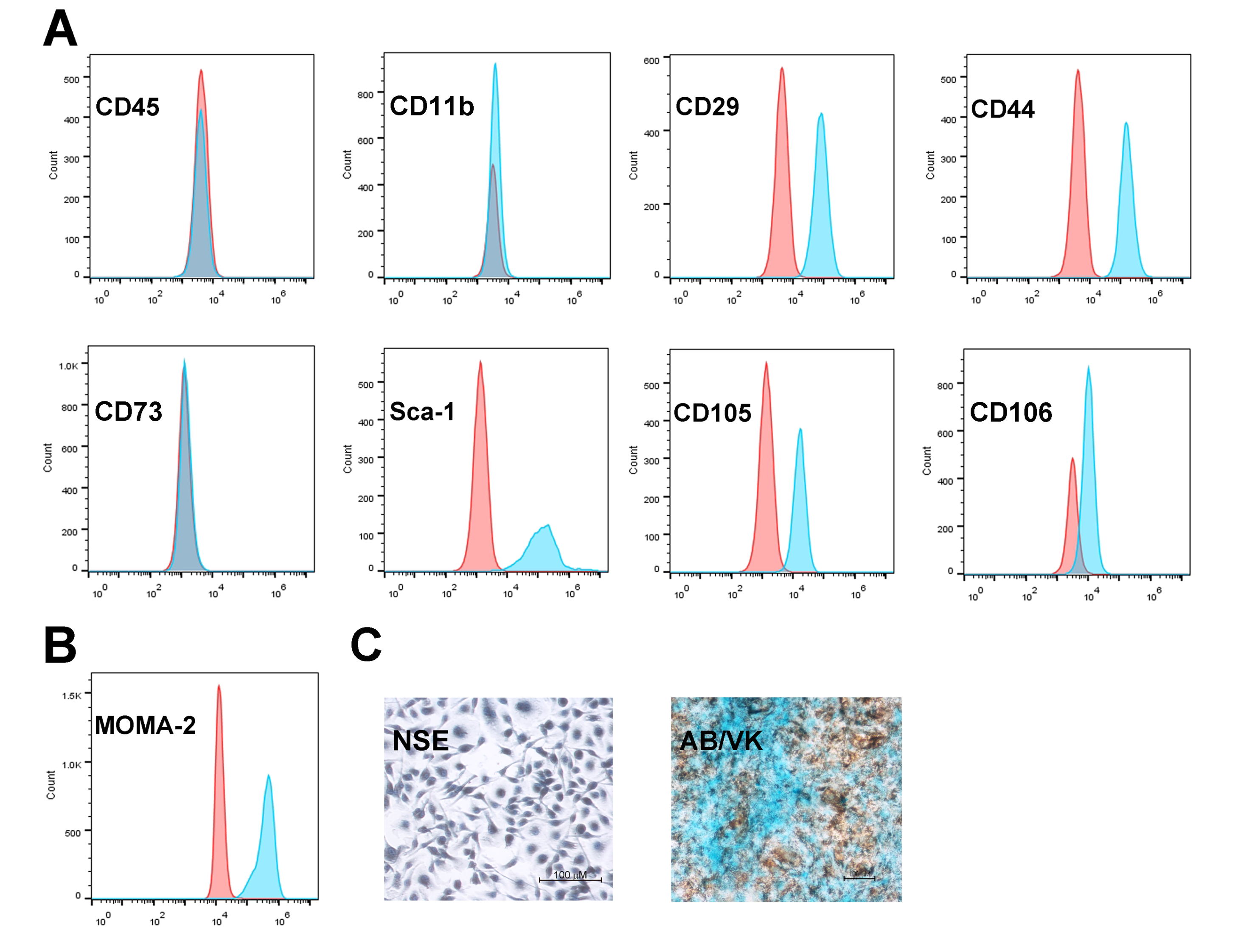

Supplement: Additional file 1: Figure S1. — Characterization of the aorta-derived progenitor (mAo) cell line and bone marrow-derived macrophage (MΦ). Flow cytometry results demonstrate expression of mesenchymal stem cell (MSC)-associated surface antigens in the mAo cell cultures (A) and the expression of the MOMA-2 monocyte/macrophage-associated intracellular antigen by the MΦ cultures (B). (C) Photomicrographs of MΦ cultures stained with non-specific esterase (NSE) in the presence of fluoride and counterstained with hematoxylin to demonstrate the nuclei. mAo cells were stained with Alcian Blue/von Kossa (AB/VK) stain after culture in chondrogenic conditions. [file 13287_2015_71_MOESM1_ESM.tiff]

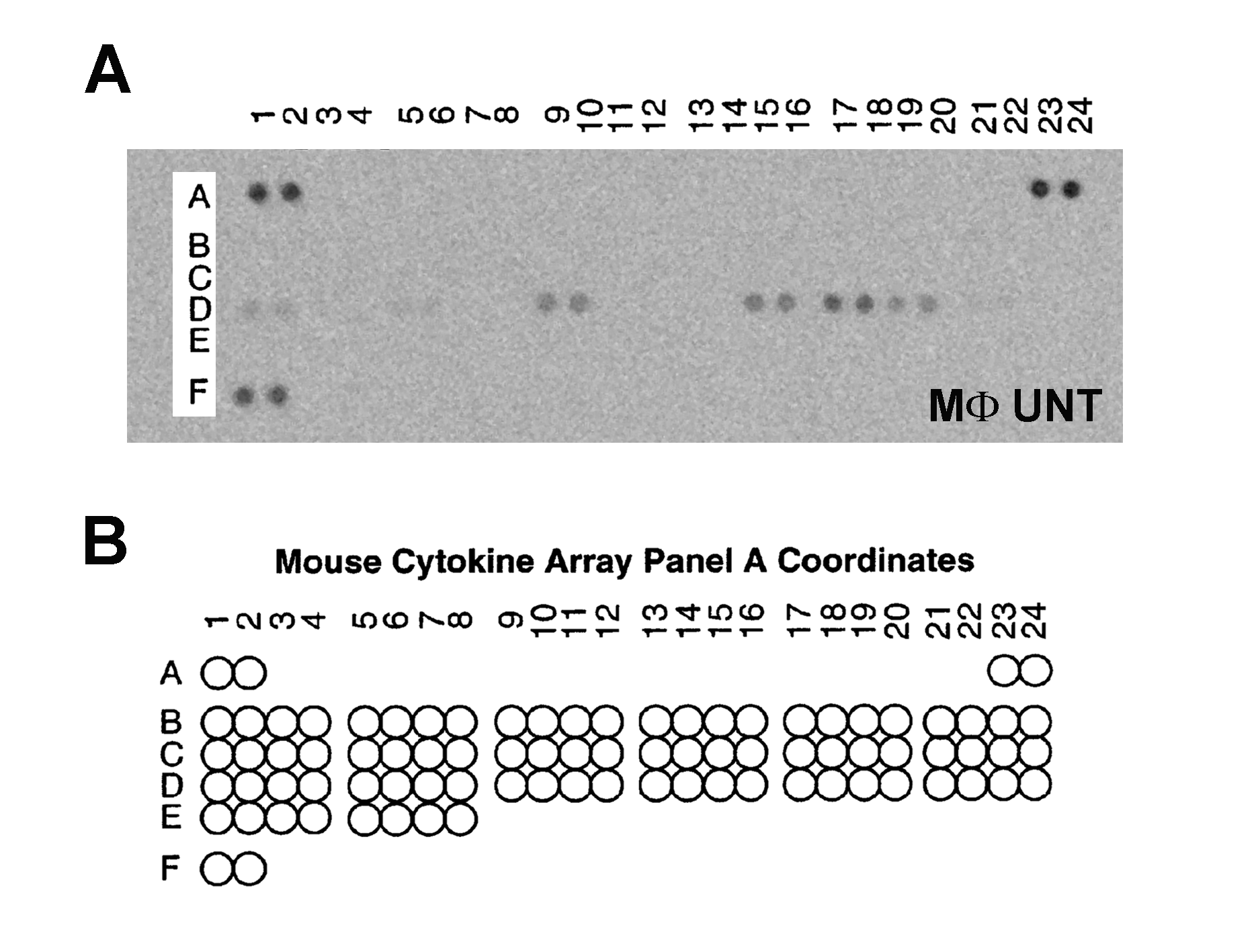

Supplement: Additional file 2: Figure S2. — Proteome Profiler cytokine/chemokine blot of untreated MΦ culture which was used as a baseline to analyze data presented in Figure 5. Densitometric data from mouse cytokine Proteome Profiler immunoblots shown in Figure 5 were compared with densitometric analysis of the untreated MΦ blot shown here (A). (B) The overlay of coordinates. The key is found in Additional file 3. MΦ, bone marrow-derived macrophage. [file 13287_2015_71_MOESM2_ESM.tiff]
