# Supplementary material for: Mouse aorta-derived mesenchymal progenitor cells contribute to and enhance the immune response of macrophage cells under inflammatory conditions
Source: Stem Cell Res Ther. 2015 Apr 14;6(1):56. doi: 10.1186/s13287-015-0071-8 (PMC4414009; doi:10.1186/s13287-015-0071-8)
Supplement: Additional file 3: — The key for the Proteome Profiler coordinates found in Additional file 2 : Figure S2. [file 13287_2015_71_MOESM3_ESM.pdf]

Refer to the table below for the Mouse Cytokine Array coordinates.

Coordinate	Target/Control	Alternate Nomenclature
A1, A2	Reference Spot	——
A23, A24	Reference Spot	——
B1, B2	BLC	CXCL13/BCA-1
B3, B4	C5/C5a	Complement Component 5a
B5, B6	G-CSF	——
B7, B8	GM-CSF	——
B9, B10	I-309	CCL1/TCA-3
B11, B12	Eotaxin	CCL11
B13, B14	sICAM-1	CD54
B15, B16	IFN-γ	——
B17, B18	IL-1α	IL-1F1
B19, B20	IL-1β	IL-1F2
B21, B22	IL-1ra	IL-1F3
B23, B24	IL-2	——
C1, C2	IL-3	——
C3, C4	IL-4	——
C5, C6	IL-5	——
C7, C8	IL-6	——
C9, C10	IL-7	——
C11, C12	IL-10	——
C13, C14	IL-13	——
C15, C16	IL-12 p70	——
C17, C18	IL-16	——
C19, C20	IL-17	——
C21, C22	IL-23	——
C23, C24	IL-27	——
D1, D2	IP-10	CXCL10/CRG-2
D3, D4	I-TAC	CXCL11
D5, D6	KC	CXCL1
D7, D8	M-CSF	——
D9, D10	JE	CCL2/MCP-1
D11, D12	MCP-5	CCL12
D13, D14	MIG	CXCL9
D15, D16	MIP-1α	CCL3
D17, D18	MIP-1β	CCL4
D19, D20	MIP-2	CXCL2
D21, D22	RANTES	CCL5
D23, D24	SDF-1	CXCL12
E1, E2	TARC	CCL17
E3, E4	TIMP-1	——
E5, E6	TNF-α	——
E7, E8	TREM-1	——
F1, F2	Reference Spot	——
F23, F24	PBS (Negative Control)	Control (-)
